# Motor Recovery After Stroke: From a Vespa Scooter Ride Over the Roman *Sampietrini* to Focal Muscle Vibration (fMV) Treatment. A 99mTc-HMPAO SPECT and Neurophysiological Case Study

**DOI:** 10.3389/fneur.2020.567833

**Published:** 2020-11-12

**Authors:** Massimiliano Toscano, Maria Ricci, Claudia Celletti, Marco Paoloni, Marco Ruggiero, Alessandro Viganò, Tommaso B. Jannini, Alberto Altarocca, Mauro Liberatore, Filippo Camerota, Vittorio Di Piero

**Affiliations:** ^1^Department of Human Neurosciences, “Sapienza” University of Rome, Rome, Italy; ^2^Department of Neurology, Fatebenefratelli Hospital, Isola Tiberina, Rome, Italy; ^3^Department of Biomedicine and Prevention, University of Rome Tor Vergata, Rome, Italy; ^4^Physical Medicine and Rehabilitation Division, Umberto I Hospital, Rome, Italy; ^5^Department of Physical Medicine and Rehabilitation, “Sapienza” University of Rome, Rome, Italy; ^6^IRCCS Fondazione Don Carlo Gnocchi, Milan, Italy; ^7^Department of Systems Medicine, University of Rome Tor Vergata, Rome, Italy; ^8^Department of Radiological, oncological and pathological Sciences - Radiometabolic Division, Umberto I Hospital, Rome, Italy

**Keywords:** stroke, focal muscle vibration, brain plasticity, spinal cord plasticity, motor recovery

## Abstract

Focal repetitive muscle vibration (fMV) is a safe and well-tolerated non-invasive brain and peripheral stimulation (NIBS) technique, easy to perform at the bedside, and able to promote the post-stroke motor recovery through conditioning the stroke-related dysfunctional structures and pathways. Here we describe the concurrent cortical and spinal plasticity induced by fMV in a chronic stroke survivor, as assessed with 99mTc-HMPAO SPECT, peripheral nerve stimulation, and gait analysis. A 72-years-old patient was referred to our stroke clinic for a right leg hemiparesis and spasticity resulting from a previous (4 years before) hemorrhagic stroke. He reported a subjective improvement of his right leg's spasticity and dysesthesia that occurred after a30-min ride on a *Vespa* scooter as a passenger over the Roman Sampietrini (i.e., cubic-shaped cobblestones). Taking into account both the patient's anecdote and the current guidelines that recommend fMV for the treatment of post-stroke spasticity, we then decided to start fMV treatment. 12 fMV sessions (frequency 100 Hz; amplitude range 0.2–0.5 mm, three 10-min daily sessions per week for 4 consecutive weeks) were applied over the quadriceps femoris, triceps surae, and hamstring muscles through a specific commercial device (Cro®System, NEMOCOsrl). A standardized clinical and instrumental evaluation was performed before (T0) the first fMV session and after (T1) the last one. After fMV treatment, we observed a clinically relevant motor and functional improvement, as assessed by comparing the post-treatment changes in the score of the Fugl-Meyer assessment, the Motricity Index score, the gait analysis, and the Ashworth modified scale, with the respective minimal detectable change at the 95% confidence level (MDC_95_). Data from SPECT and peripheral nerve stimulation supported the evidence of a concurrent brain and spinal plasticity promoted by fMV treatment trough activity-dependent changes in cortical perfusion and motoneuron excitability, respectively. In conclusion, the substrate of post-stroke motor recovery induced by fMV involves a concurrently acting multisite plasticity (i.e., cortical and spinal plasticity). In our patient, operant conditioning of both cortical perfusion and motoneuron excitability throughout a month of fMV treatment was related to a clinically relevant improvement in his strength, step symmetry (with reduced limping), and spasticity.

## Introduction

Stroke is the leading cause of long-term disability (1) with reduced mobility in more than half of stroke survivors aged 65 and over (2).

A focal brain lesion resulting from stroke may trigger maladaptive structural and functional changes in perilesional and remote regions (3–5), thereby resulting in incomplete motor recovery. Thus, to evaluate which could be the most effective training protocol for the rehabilitation of a paretic limb, there is a growing interest toward the attempts to condition the dysfunctional cerebral structures and pathways throughout non-invasive central and peripheral stimulation techniques (NIBS), as seen for others neurological diseases (6–10).

Focal repetitive muscle vibration (fMV) is a safe and well-tolerated intervention, easy to perform at the bedside, and able to promote motor recovery both in acute (11) and chronic stroke patients (12–15).

Despite the great number of recent works, the mechanism underlying motor improvement after fMV in stroke patients is not completely understood yet. Given that a repeated sensory input is one of the most effective modulators of cortical motor and somatosensory structures (16), it has been suggested that fMV can produce substantial neurophysiological changes both at a cortical and at a peripheral level (12, 17), probably throughout long-term depression-like plasticity mechanisms (18).

Here we describe the concurrent cortical and spinal plasticity induced by fMV in a chronic stroke survivor, as assessed with 99mTc-HMPAO SPECT, peripheral nerve stimulation, and gait analysis. We also focused on the relationship between the observed multisite plasticity and patient's improvement in different motor aspects (i.e., strength, gait, and spasticity) after fMV treatment.

## Case Study—From a *Vespa* Scooter Ride Over the Roman Sampietrini to Focal Muscle Vibration (fMV)

A 72-years-old patient was referred to our stroke clinic for a right leg hemiparesis and spasticity resulting from a previous (4 years before) hemorrhagic stroke. The neurological examination also showed right-sided severe hypoesthesia and dysesthesia associated with tactile extinction.

He has been treated with Tizanidine (up to 36 mg/day) and physiokinesitherapy (1-h daily session, 3 days a week) with no further clinical improvements in the past year. No other vascular risk factors were known but hypertension. A computer tomography (CT) scan showed the results of the known cerebral hemorrhage involving the left thalamus and the internal capsule.

In a sort of “Roman Holidays” movie set, our patient anecdotally reported subjective clinical improvement in spasticity and dysesthesia of the right leg that occurred after a 30-min ride as a passenger on a *Vespa* scooter. The ride had taken place on a paved road by Roman Sampietrini (i.e., cubic-shaped cobblestones) which produced many vibrations.

Taking into account both the patient's anecdote and the current guidelines which recommend focal muscle vibration (fMV) for the treatment of post-stroke spasticity (14), we then decided to start fMV treatment.

### fMV Treatment

A total of 12 fMV sessions (3 sessions per week for 4 consecutive weeks) were carried out by a trained physiatrist. Each daily session consisted of three 10-min treatment, interspersed with a 1-min break (11).

Low-amplitude fMV (frequency 100 Hz; amplitude range 0.2–0.5 mm) was applied over the quadriceps femoris, triceps surae, and hamstring muscles through a specific commercial device (Cro®System, NEMOCOsrl). During the fMV treatment, the subject was required to maintain a voluntary steady contraction of the target muscle at 20% of the maximal voluntary contraction (MVC), as assessed by visual EMG feedback (12, 19). During the intervals (1 min), fMV was interrupted and the subject was requested to relax the muscle. fMV was delivered during mild voluntary contraction because it was shown that voluntary muscle activity increases response to vibration, probably through fusimotor co-activation and subsequent increase in spindle discharge (18, 20). Moreover, the prolonged activation of the descending volley, induced by the ongoing muscle contraction with ascending volleys, might explain the changes in corticomotor excitability of the target muscle (19).

### Clinical Assessment

**Figure 1:**
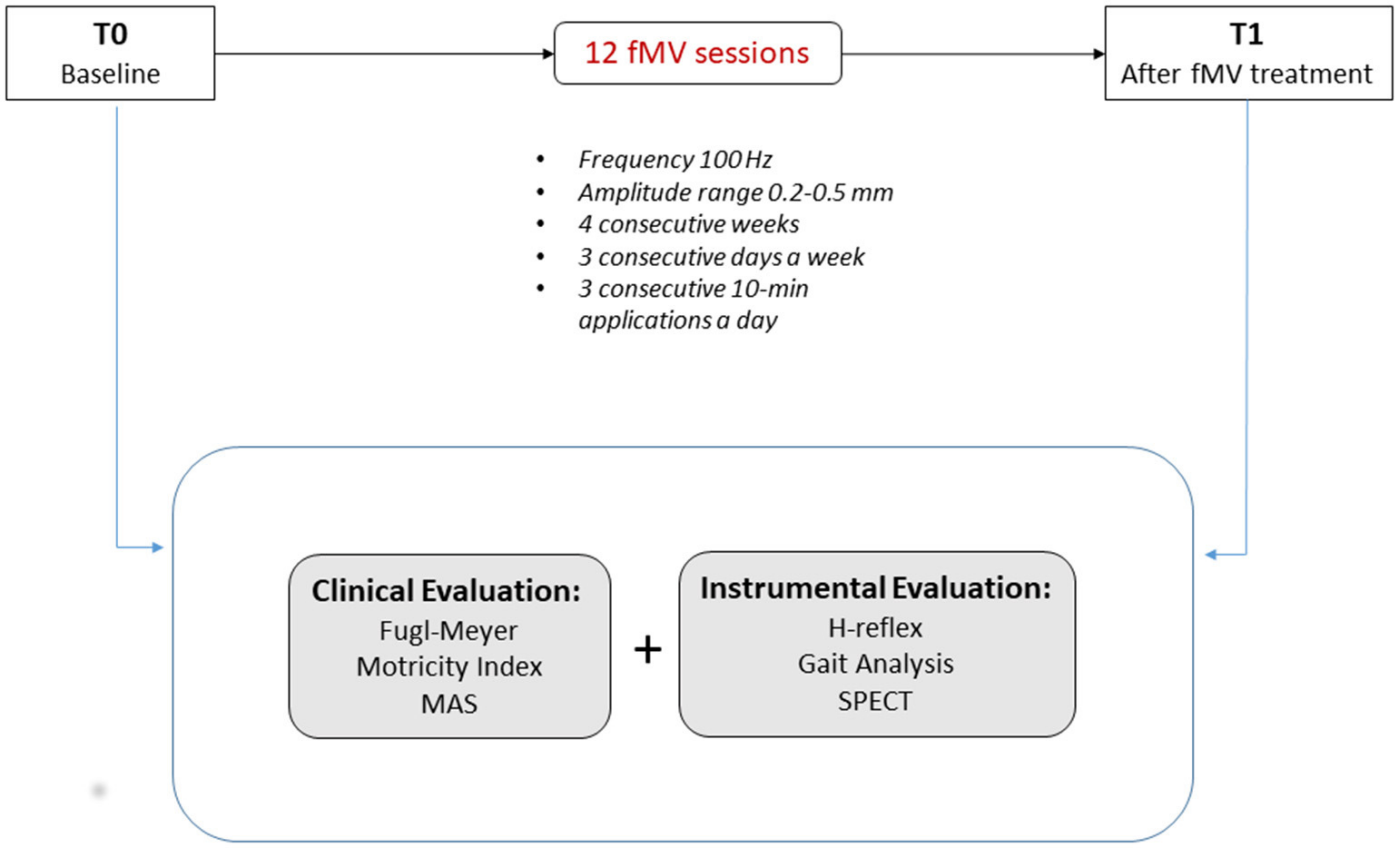
Study flow chart—clinical and instrumental evaluation was performed before (T0) the first fMV session and 7 days after the last one (T1).

To examine fMV effects, a standardized clinical and instrumental evaluation was performed before (T0) the first fMV session and 7 days after (T1) the last one as follows (see Figure 1 for the study flow chart).

Motor and functional performance were evaluated by using both the lower limb subscale of Fugl-Meyer assessment (FMA) (21–23) and the Motricity Index (MI) (24). FMA subscale for the lower limb examines movement, coordination, and reflex action of the hip, knee, and ankle in the supine, sitting, and standing positions. Each item is scored on a 3-point scale (0, cannot perform; 1, partially performs; 2, performs fully). The score range is 0 to 34, with higher scores indicating better lower limb motor performance. The minimal detectable change at the 95% confidence level (MDC_95_) is 4-point (25).

MI examines hip flexion, knee extension, and ankle dorsiflexion (3 items for each side). The total score ranges from 0 (complete paresis) to 100 (normal strength), with an MDC_95_ is 12.92 (26).

Spasticity was assessed with the Ashworth scale modified by Bohannon and Smith (27) (MAS), which measures resistance during passive soft-tissue stretching scoring from 0 (no increase in muscle tone) to 4 (affected part(s) rigid in flexion or extension). A one-point decrease reflects a clinically significant improvement at the 95% confidence level MDC_95_ (28).

### Gait Analysis

As regards the instrumental evaluation, we performed a gait analysis to evaluate cadence (step/min), velocity, right, and left stride length. We adopted the inertial measurement unit (IMU), which includes a triaxial accelerometer (16 bits/axis, up to 1,000 Hz) with multiple sensitivity (± 2, ± 4, ± 8, ± 16 g), a triaxial 13-bit magnetometer (± 1,200 μ T, up to 100 Hz), and a triaxial gyroscope (16 bits/axis, up to 8,000 Hz) with multiple sensitivity (± 250, ± 500, ± 1,000, ± 2,000 °/s). The MDC_95_ for gait analysis' spatiotemporal, kinematic, and kinetic measurements can be found in Geiger et al. (29).

### Peripheral Nerve Stimulation

Electromyography (EMG) was performed on the tibial nerve (mixed nerve), using stimuli of increased intensity to evaluate H-reflex, M-reflex, and their ratio H/M. The size of the H-reflex and M-response was defined as the peak-to-peak amplitude of the EMG record. To record the electrical activity of the soleus muscle, both surface and intramuscular EMG were used according to the standardized registration technique proposed by Gorkem et al. (30).

### SPECT Imaging

**Figure 2:**
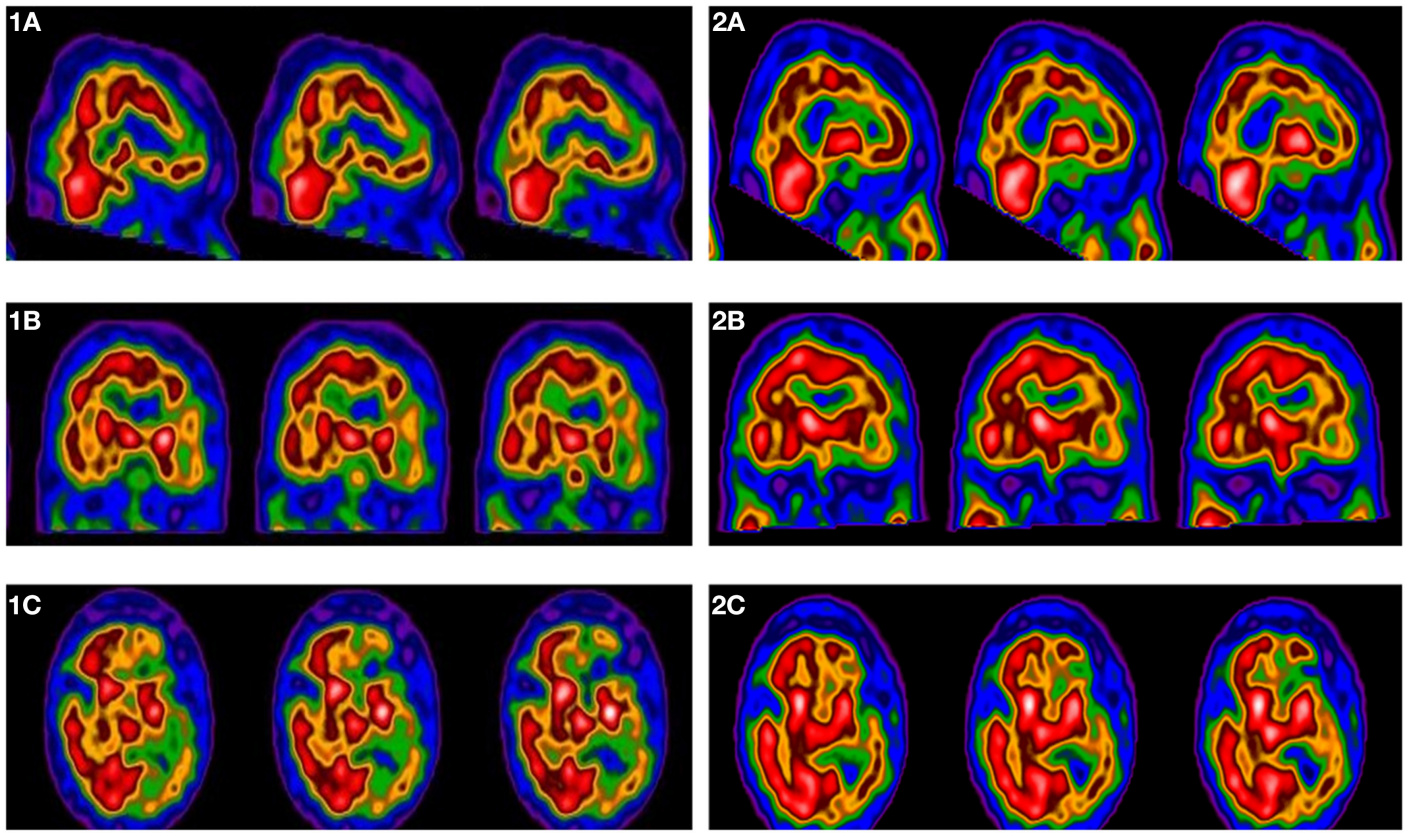
^99m^Tc-HMPAO brain perfusion SPECT scan at baseline in sagittal **(1a)**, coronal **(1b)**, and transaxial **(1c)** reconstruction; ^99m^Tc-HMPAO perfusion brain SPECT scan after fMV treatment in sagittal **(2a)**, coronal **(2b)** and transaxial **(2c)** reconstruction. SPECT images show an increased ^99m^Tc-HMPAO distribution rate after fMV treatment in left frontal region (**1a** vs. **2a**), in left parietal region (**1b** vs. **2b)**, in left occipital region (**1c** vs. **2c**).

99mTc-HMPAO Single Photon Emission Computed Tomography (SPECT) was performed to evaluate functional cortical reorganization and perfusion, assessed by the evaluation of 99mTc-HMPAO distribution. The blood perfusion in the left and right cerebral hemispheres was assessed in rest status both at baseline and after treatment (Figure 2). The patient was asked to avoid caffeine, smoke, alcohol, and psychotropic drug that can affect the cerebral blood flow.

The imaging data were evaluated by 2 experienced operators that performed a qualitative analysis. The semi-quantitative analysis was carried out using the NeuroGam software. NeuroGam software (Segami-Corporation, http://www.segamicorp.com/) allows statistical analysis methods for automated diagnosis of brain perfusion SPECT images and can be used to investigate brain perfusion objectively and easily. This software applies an affine anatomical co-registration by blocks of data defined in the Talairach space. The Talairach technique normalized brain cortical perfusion measurements to the perfusion of the cerebellum (31).

NeuroGam was applied to the reoriented and reconstructed data as follows: we reoriented the three-dimensional volume of the brain study defining a line that fits the inferior pole of the occipital lobe and the inferior edge of the frontal lobe. Then, we defined a line above the interhemispheric fissure and automatically orienting this line in the vertical plane to avoid the lateral deviations. After the reorientation of the image, we defined the intermediate level of the pons and anterior plane of the temporal lobes. Therefore, data were processed analyzing ten regions of interest (ROI), corresponding to the entire cortex of the hemisphere, to the parietal, temporal, frontal, and occipital regions, all analyzed with respect to the contralateral region. Data were expressed as maximum, minimum, and mean standard deviation of count rate registered.

The semiquantitative evaluation of brain perfusion in cortical areas of both hemispheres has been automatically performed by the software which automatically calculates the mean and standard deviation of each cortical region's perfusion level (32). The reconstructed data have been also compared to the normal database available for the NeuroGam analysis. Further SPECT imaging's technical details can be found in Frantellizzi et al. (33) Mancini et al. (34).

### Ethics Statement

Written informed consent was obtained from the patient both to participate in the study and for the publication of any potentially identifiable images or data included in this article. The study was conducted in accordance with the Declaration of Helsinki Ethical Principles and Good Clinical Practices and was approved by the local ethics committee (Ethics Committee of “Sapienza,” University of Rome—Ref. No. 3661).

## Results

After fMV treatment (T1), the patient reported a substantial clinical improvement in his leg's spasticity and dysesthesia, as well as a subjective feeling of “more fluent walking,” that gradually started within the first week of fMV treatment and lasted until T1 evaluation (i.e., 7 days after the end of fMV treatment).

The neurological examination performed at T1 showed an increase in the FMA global score compared to baseline (27 vs. 23, respectively). Namely, we found an improvement in the hip flexion, knee flexion/extension, and ankle dorsiflexion FMA's items. The MI score increased from 76 at T0 to 91 at T1. After treatment, we also found a reduction of the hypertonus as assessed with MAS (T0 vs. T1 MAS score: 2 vs. 1).

The gait analysis was performed by assessing the cadence, speed, and length of the step. When comparing left and right stride length, we found a higher right-left symmetry after treatment (T1 left stride length vs. right stride length: 0.49 ± 0.08 m vs. 0.5 ± 0.09 m) when compared to baseline (T0 left stride length vs. right stride length: 0.43 ± 0.15 m vs. 0.56 ± 0.11 m). We also found a slight decrease in both step cadence (T0 vs. T1: 75.84 ± 16.19 vs. 72.86 ± 16.31 step/min) and mean velocity (T0 vs. T1: 0.66 ± 0.14 vs. 0.61 m/s).

H-reflex amplitude decreased from 1 mv (T0 value) to 0.1 mv (T1 value). The H/M ratio expressed as a percentage was 81.9% at T0, whereas at the end of fMV treatment was 25.9%. Complete data from H-reflex analysis are reported in Table 1.

**Table 1:** Characteristics of the H-reflex elicited through stimulation of the tibial nerve.

	**M max (Resp. No.)**	**H max (Resp. No.)**	**H latency (ms)**	**H amplitude (mV)**	**H/M ratio**
Tibial nerve (T0)	24	19	14.3	1	81.9%
Tibial nerve (T1)	16	13	19.05	0.1	25.9%

*(H latency, H amplitude, H/M ratio, maximal M wave (M max), and HR (H max)). See the text for details*.

SPECT data comparing T0 and T1 assessments of cerebral perfusion are shown in Table 2. The qualitative analysis showed a more homogenous perfusion pattern after treatment, with a global improvement of perfusion in the affected hemisphere. In the semiquantitative analysis, the mean standard deviation count rate of 99mTc-HMPAO distribution in the whole brain was higher after treatment compared to baseline (T0 vs. T1: −1.9 vs. −1.6) suggesting a global improvement of blood perfusion.

**Table 2:** SPECT–Data comparing T0 and T1 assessments of cerebral perfusion.

**ROI**	**Baseline (T0)**			**After fMV treatment (T1)**		
	**Max s.d**.	**Min s.d**.	**Mean s.d**.	**Max s.d**.	**Min s.d**.	**Mean s.d**.
Cort. Right	5	−5	−1.2	5	−5	−1.5
Cort. Left	3.6	−5	−3.3	5	−5	−2.8
Front. Right	5	−5	−1.4	5	−5	−1.7
Front. Left	3.6	−5	−3.1	5	−5	−2.9
Occ. Right	4.4	−5	0.5	5	−5	1.1
Occ. Left	1.5	−5	−2.7	3.7	−5	−1.6
Par. Right	3.6	−5	−0.4	5	−5	0
Par. Left	3.2	−5	−2.6	5	−5	−1.7
Temp. Right	4.8	−5	−1.5	4.9	−5	−2.1
Temp. Left	5	−5	−3.6	3.8	−5	−3.2
Whole	5	−5	−1.9	5	−5	−1.6

*To define the regions of interest (ROI), NeuroGam software (Segami-Corporation, http://www.segamicorp.com/) was applied to the reoriented and reconstructed three-dimensional brain volume data (see the text for details). Therefore, data were processed analyzing ten ROI, corresponding to the entire cortex of the hemisphere, to the parietal, temporal, frontal, and occipital regions, all analyzed with respect to the contralateral region. Data are expressed as maximum, minimum, and mean standard deviation of count rate registered*.

The distribution of 99mTc-HMPAO after fMV also suggested a global improvement of the mean rate of perfusion reported in areas affected by stroke. Namely, the distribution of 99mTc-HMPAO was higher at T1 in comparison to baseline both in the entire left cortex (T0 vs. T1: −3.3 vs. −2.8), and in single brain regions as the left frontal lobe (T0 vs. T1: −3.1 vs. −2.9), left occipital lobe (T0 vs. T1: −2.7 vs. −1.6), left parietal lobe (T0 vs. T1: −2.6 vs. −1.7), and left temporal lobe (T0 vs. T1: −3.6 vs. −3.2). The distribution of the radiolabeled compound after treatment, considering the semiquantitative nature of Neurogam analysis, was reduced in the right cortex compared to baseline (T0 vs. T1: −1.2 vs. −1.5).

## Discussion

Since the last two decades, the attempt to condition the mechanisms underlying plasticity throughout non-invasive central and peripheral stimulation techniques has been pivotal to enhance recovery of locomotion in many neuromuscular disorders and stroke as well.

To test the hypothesis that fMV can produce changes at a cortical and spinal level simultaneously and that these changes are concurrently responsible for the post-stroke motor improvement, we analyzed in the same patient the changes in brain perfusion elicited by spinal H-reflex open conditioning throughout a month of fMV treatment.

In this regard, there is no agreement in the literature about the number of fMV sessions required for motor improvement to be very effective. Since the amount of the induced change grows gradually as conditioning trials continue over subsequent days and weeks (35, 36) we decided to carry out a total of 12 fMV sessions (3 sessions per week for 4 consecutive weeks), thereby extending the classic one-week treatment (overall 3 sessions) (13, 19, 37).

### fMV-Induced Brain Plasticity

Our data showed an fMV-induced increase of the 99mTc-HMPAO distribution rate in the left hemisphere, suggesting a global improvement of the mean rate of perfusion in the affected hemisphere after treatment. This confirms that the repeated muscle vibration produces a repeated sensory input that reaches, via Ia fiber afferent input, both the SI and M1 directly (38–41), thereby leading to an improvement of affected limb's motor and functional performance through an intrinsic plasticity-related mechanism (13, 42, 43).

Interestingly, we also found a post-treatment reduction in the distribution of the radiolabeled compound in the right cortex compared to baseline. A possible explanation is that fMV can reduce abnormalities of both the corticospinal excitability and the intracortical inhibitory systems in chronic stroke patients (12), trough modulating the post-stroke altered balance of excitatory and inhibitory influences within the motor network.

Due to the semiquantitative nature of the analysis performed, the 99mTc-HMPAO distribution in the right cortex is influenced by the contralateral compound distribution and, therefore, the reduction of 99mTc-HMPAO distribution in the unaffected hemisphere may reflect the perfusion improvement of the affected one.

Thus, we cannot confirm that the improvement of the mean rate of perfusion in the affected hemisphere directly caused the decrease of perfusion in the unaffected hemisphere through the modulation of the transcallosal inhibition. However, the fMV treatment showed a plasticity-dependent increase of perfusion as a marker of reactivation of functionally impaired circuits within the motor network (44).

### fMV-Induced Spinal Cord Plasticity

Operant conditioning of the H-reflex (i.e., the electric analog of the spinal stretch reflex) has widely considered as a valid paradigm for investigating the plasticity induced in the human spinal cord (36). Evidence from studies on healthy subjects (17, 18) suggests that fMV is able to promote spinal plasticity by inducing a decrease of the H-reflex. Thus, H-reflex operant conditioning techniques, as fMV, may represent a new approach for enhancing functional recovery.

In our patient, we observed a reduction in the amplitude of H-reflex from T0 to T1. The change in H-reflex was strongly related to fMV treatment (i.e., activity-dependent) since it has been demonstrated that a sustained voluntary muscle contraction is not able to induce changes of the H-reflex (18). Thus, fMV induces a change in the intrinsic spinal cord properties, probably consisting of an activity-dependent decrease of the intrinsic motoneuron excitability (17) throughout long-term depression-like (LTD-like) plasticity.

A decreased motoneuronal excitability was further confirmed in our patient by the reduction in the H/M ratio from 81.9% at T0 to 25.9% at T1. Given that H max represents an indirect estimate of the number of motoneurons (MN) being recruited, and the M max represents the entire motoneuron pool, the H/M ratio can be interpreted as the proportion of the entire MN pool capable of being recruited (45).

Taken together, data from SPECT and peripheral nerve stimulation support the evidence of a concurrent brain and spinal plasticity promoted by fMV treatment trough activity-dependent changes in cortical perfusion and motoneuron excitability, respectively.

### Motor Recovery

After describing the intrinsic changes in the brain and the spine induced by fMV, we aimed to test if these changes were related to an improvement in our patient's motor performance. Thus, we analyzed his strength, gait, and spasticity at 7 days after the end of fMV treatment.

To understand if the patient experienced a clinically relevant change after fMV treatment, we compared the post-treatment changes in the score of each scale with the respective MDC_95_, which is the smallest real difference that can be detected by a scale at the 95% confidence level beyond measurement error. MDC_95_ represents the minimal amount of change in the score that must occur to be sure that the change in score is not simply attributable to measurement error and helps clinicians to understand data in the clinical setting also at the individual level (28).

Overall, we observed a clinically relevant motor and functional improvement after fMV treatment. We found an increase in the T1 FMA global score compared to baseline (27 vs. 23, respectively, MDC_95_:4), with an improvement in the hip flexion, knee flexion/extension, and ankle dorsiflexion. MI score increased from 76 at T0 to 91 at T1 (MDC_95_:12.92).

Data from gait analysis showed that, beyond a clinically relevant change in both the right and left stride length (T0/T1 right stride length: 0.56/0.5 m; T0/T1 left stride length: 0.43/0.49 m. MDC_95_:0.06 m), right and left values became similar after treatment, that indicates a recovery of the step symmetry with reduced limping. Although operant conditioning can be a useful therapeutic tool for enhancing locomotion in patients with spinal cord injuries, the mechanism underlying the fMV-induced effects on the symmetry of the step is still debated.

It is well-known the role of fMV in reducing spasticity when applied to the spastic muscles of hemiplegic limb in post-stroke patients (13, 46, 47). Thus, we hypothesized that the observed findings are ascribable to the effects of fMV stimulation, which would result in better motor control of the affected limb and inhibition of pathological motor patterns through a reduction of the hypertonicity degree. This effect was confirmed in our patient, since we found a clinically relevant activity-dependent reduction of the quadriceps femoral muscle's hypertonus, as assessed with the MAS (MDC_95_:1).

A limitation of the present study is that we use MAS to assess spasticity without considering the muscular viscoelasticity, which is another component of passive resistance to stretch of muscles. Anyway, MAS is widely considered a standard method for the assessment of spasticity in clinical settings. Moreover, several factors may influence the distribution of 99mTc-HMPAO, the reproducibility of the SPECT studies, and the semi-quantitative analysis of the data. Further studies using higher-resolution techniques (i.e., fMRI or quantitative EEG) are needed to confirm this datum.

## Conclusions

The substrate of post-stroke motor recovery induced by fMV involves a concurrently acting multisite plasticity (i.e., cortical and spinal plasticity). In our patient, operant conditioning of both cortical perfusion and motoneuron excitability throughout a month of fMV treatment was related to an clinically relevant improvement in his strength, step symmetry (with reduced limping), and spasticity.

## Data Availability Statement

The original contributions presented in the study are included in the article/supplementary materials, further inquiries can be directed to the corresponding author/s.

## Ethics Statement

The study involving human participants was conducted in accordance with the Declaration of Helsinki Ethical Principles and Good Clinical Practices and was approved by the local ethics committee (Ethics Committee of “Sapienza”, University of Rome). Written informed consent was obtained from the patient both to participate in the study and for the publication of any potentially identifiable images or data included in this article.

## Author Contributions

MT: study design and overview and manuscript preparation. MRi: SPECT data analysis and manuscript preparation. CC: fMV execution and data analysis. MP: peripheral nerve stimulation and gait analysis. MRu: clinical evaluation and data analysis. AV: literature review and manuscript review. TJ: literature review and ethics committee. AA: clinical scales' implementation and review. ML: SPECT overview and manuscript review. FC: fMV overview and manuscript review. VD: data interpretation and manuscript review. All authors: contributed to the article and approved the submitted version.

## Conflict of Interest

The authors declare that the research was conducted in the absence of any commercial or financial relationships that could be construed as a potential conflict of interest.
